# Fluid Intelligence Emerges from Representing Relations

**DOI:** 10.3390/jintelligence10030051

**Published:** 2022-08-02

**Authors:** Adam Chuderski

**Affiliations:** Cognitive Science Department, Institute of Philosophy, Jagiellonian Univeristy in Krakow, PL-31007 Kraków, Poland; adam.chuderski@uj.edu.pl

**Keywords:** fluid intelligence, relation, binding

## Abstract

Based on recent findings in cognitive neuroscience and psychology as well as computational models of working memory and reasoning, I argue that fluid intelligence (fluid reasoning) can amount to representing in the mind the key relation(s) for the task at hand. Effective representation of relations allows for enormous flexibility of thinking but depends on the validity and robustness of the dynamic patterns of argument–object (role–filler) bindings, which encode relations in the brain. Such a reconceptualization of the fluid intelligence construct allows for the simplification and purification of its models, tests, and potential brain mechanisms.

## 1. Introduction

*Intelligence* (*general cognitive ability*) constitutes one of the central constructs in psychology, originating from the late nineteenth century (e.g., Galton 1883; Gilbert 1894). Its main purpose is twofold. On the one hand, intelligence research attempts to explain the enormous variability in intellectual powers found in any human population (and, recently, even in dogs; Arden and Adams 2016). On the other hand, this research needs to explain the considerable stability of these powers in individuals, meaning that their scores on one kind of intellectual test strongly correspond to their scores on other kinds of test (a phenomenon called *positive manifold*). The structure of human intelligence (various general and specific abilities and their mutual relations) as well as its predictive power have been a topic of vivid debate (e.g., Carroll 1993; Horn and Cattell 1966; Jäger 1982; Kovacs and Conway 2016; Spearman 1927; Thomson 1919; Thurstone 1938; Van Der Maas et al. 2006; Vernon 1964), suggesting that intelligence constitutes a complex entangled multilevel construct (McGrew 2009) reflecting the brain structure and function (Deary et al. 2010; Haier 2016).

However, as a comprehensive measurement of intelligence with diverse batteries of tests (knowledge use, verbal and memory skills, visual and auditory processing, mental speed, etc.) is not feasible, the research on cognitive abilities frequently focuses on fluid intelligence (Cattell 1963), also called fluid reasoning (Carroll 1993) or reasoning ability (Kyllonen and Christal 1990). According to the Cattell–Horn–Carroll model of abilities (McGrew 2009), fluid intelligence has been best-reflected by novel reasoning problems solved in a deliberate and controlled way, which cannot be automatized. In this model, fluid intelligence comprises at least three narrow abilities, namely deductive (called also general sequential), inductive, and quantitative reasoning. Whether these three abilities rely on separable processes, or stem from a single mechanism, such as mental model construction and verification (Johnson-Laird 2006) or Bayesian inference (Oaksford and Chater 2007), remains an open question; however, the fact that deductive and inductive subfactors typically correlate almost perfectly (Wilhelm 2005) suggests the latter case. Other authors also differentiated content types of fluid intelligence, specifically its verbal, numerical, and figural facets (Jäger 1982). Evidence is stronger for content than process facets (Lakin and Gambrell 2012; Schulze et al. 2005), suggesting that a specific test content involves also a respective ability beyond fluid reasoning (e.g., figural and spatial tasks may also require visual processing ability). In practice, content is frequently confounded with task type, as most of deductive tests are verbal, most of the inductive tests are figural–spatial, and quantitative tests by definition need to rely on numerical material (Wilhelm 2005). Figural inductive tests seem to be administered most. 

The rationale for operationalizing cognitive ability as fluid intelligence is threefold at least. First, compared to all other abilities, fluid intelligence most strongly loads general intelligence factor (*g*), with loadings reaching unity (Arendasy et al. 2008; Gustafsson 1984; Kan et al. 2011), at least in homogenous samples (Kvist and Gustafsson 2008). Second, fluid intelligence tests display especially high validity and reliability, which have been refined for a century (Cattell 1949; Raven 1938). Third, considerable efforts have been devoted to understanding the processes captured by fluid reasoning tests (e.g., Carpenter et al. 1990). Therefore, even though in principle studying only fluid intelligence could narrow our understanding of a broader concepts of intellect, in this paper I will focus on fluid intelligence as a very feasible and valid way to study general human cognitive ability.

The goal of this work is to provide a selective discussion of existing knowledge on the cognitive mechanisms potentially responsible for individual differences in fluid intelligence. Based on available evidence and plausible models, I propose that effective solving of a fluid intelligence task in the mind can amount to representing the key task relation(s) in a valid and robust way, by linking respective elements with the roles that these elements play in the relation(s), using flexible patterns of bindings. 

## 2. Psychometric Studies on Fluid Intelligence

Just after the advent of cognitive psychology—a discipline devoted to the understanding of architecture and mechanisms of human cognition using precise experimentation (Neisser 1967)—researchers began to search for elementary cognitive processes (ECPs)—the ones captured by tasks lasting from hundreds of milliseconds to several seconds—that could predict intelligence (Hunt et al. 1975; Jensen and Munro 1979). Initial efforts comprised applying a selected cognitive task (e.g., a short-term memory task, a visual search task, a forced choice task) and correlating its scores with the scores on a selected intelligence test (e.g., Raven’s Progressive Matrices, Cattell CFT-3, Wechsler’s Adult Intelligence Scale). Typically observed correlations were relatively low (10% of variance shared), very rarely approaching 50% variance (e.g., Neubauer 1990). Around the 1990s, progress in psychometrics indicated that the use of single tasks strongly underestimated the relationships between ECPs and intelligence. The task batteries tapping into a construct, by means of various tasks as well as latent variable modeling, allowed for achieving higher reliability by filtering out unwanted sources of variance (e.g., method-specific). This research identified several ECPs related with intelligence.

Processing speed, measured by response times on forced choice tasks, by performance on clerical tasks, and by inspection time, has been considered as a promising candidate from the 1970s onwards (Jensen and Munro 1979; Salthouse 1993; see Jensen 2006). However, subsequent meta-analyses suggested that processing speed indices moderately correlate with intelligence (Doebler and Scheffler 2016; Schubert 2019; Sheppard and Vernon 2008), and studies showed that this moderate contribution is fully mediated by other factors (Conway et al. 2002; Jastrzębski et al. 2021; Troche and Rammsayer 2009; for a defense of the speed account, see Schubert and Frischkorn 2020). 

Since the 1980s (e.g., Stankov 1983), various forms of attention have been associated with intelligence, but reported correlations with intelligence were also low (Schweizer 2010; Unsworth et al. 2010). Even though not predicted by attention functioning per se, in the 1990s intelligence was linked with control over attention (attention control), understood as the mechanism responsible for focusing on task-relevant information, while blocking distraction and interference (Engle et al. 1999), or more generally as goal-related processing (Diamond 2013). Although some studies reported significant correlations between attention control and fluid intelligence (Unsworth et al. 2014), this line of research noted problems with the reliability and validity of presumed measures of attention control (Draheim et al. 2021; Hedge et al. 2018). Moreover, correlations with intelligence pertained to a single test of attention control (the anti-saccade task) and barely to its other established measures (Chuderski et al. 2012; Frischkorn and von Bastian 2021; Rey-Mermet et al. 2019). 

Moreover, in the 1990s, interest in sensorimotor discrimination, initially considered at the dawn of intelligence research (Galton 1883), was revived (Deary 1994). However, despite early positive evidence that the efficiency of discriminating stimuli in visual, auditory, and even tactile modalities can predict intelligence (Deary et al. 2004; Li et al. 1998; Meyer et al. 2010), finally its contribution was low and fully mediated by working memory (Jastrzębski et al. 2021; Troche et al. 2014). 

Working memory capacity (WMC), considered in intelligence research around that time, is reflected in the number of briefly maintained and then recalled/recognized items; typically, these are items difficult to articulate or presented under additional load and/or encoding requirements. WMC appears to be the strongest predictor of intelligence (e.g., Colom et al. 2008; Engle et al. 1999), explaining from two- to three-quarters of its variance (Oberauer et al. 2005). Other types of memory, such as long-term (Unsworth 2019) and associative memory (Williams and Pearlberg 2006), yielded much weaker contributions, so WMC seems special for intelligence. 

One could summarize the existing correlational research on ECPs and fluid intelligence as bringing us closer to understanding the cognitive mechanisms underlying the latter construct. Specifically, some potential mechanisms (bare attention and stimulus discrimination) can be discarded, others (processing speed, attention control, and possibly long-term memory) should still be considered but require additional research, whereas active maintenance of and access to task-related information, as reflected by WMC, looks as if it is a fundamental mechanism for fluid intelligence. While I agree that correlational studies provided a multitude of results, I see at least three problems in their unequivocal interpretation, and thus applicability for an advancement of fluid intelligence theory.

## 3. Theoretical Limits of Psychometric Studies

First, the strength of relationships between fluid intelligence and its relatively strong predictors, especially working memory, might have been overestimated. For instance, measurement of distinct cognitive abilities may confound their true relations with some contextual factors (e.g., motivation, boredom, testing settings, etc.) that can boost shared variance. In line with this, when Chuderski and Jastrzębski (2018) controlled motivation, anxiety, openness to experience, and age in a relatively large psychometric study, an initially almost-isomorphic relationship between broad working memory factor and fluid intelligence factor equaling *r* = .94 dropped to *r* = .74, that is, as much as 38% of initially shared variance could actually be explained by other factors. 

Moreover, although most fluid intelligence tests were initially designed as power tests, typical testing conditions (large samples, long procedures) entice researchers to cut original administration times. That only affects the tests’ reliability a little, but may hugely alter their validity (Lu and Sireci 2007), for example, increasing the role of attention and immediate memory, while decreasing the impact of longer-lasting processes such as counterexample construction, solution verification, and schema learning—all identified as crucial stages of deductive and inductive reasoning (Holyoak 2012; Johnson-Laird 2006). Moreover, under high time pressure, the late test items, which require the most advanced reasoning, are rarely attempted by participants (Estrada et al. 2017). Indeed, when Chuderski (2013) manipulated the administration time of two intelligence tests, their shared variance with a working memory factor dropped from 100% for strictly limited time to only 36% for virtually unlimited time (see also Chuderski 2015a; Ren et al. 2018). Under strict time pressure, the variance in the most difficult test items was virtually null. When the truly shared variance between fluid intelligence and working memory in fact falls below 50% instead of approaching unity, then the connection of these two constructs, even though still substantial, is no longer close-to-perfect and leaves room for alternative explanations of the fluid intelligence underpinnings.

Second, no cognitive task developed so far can capture a unique “elementary cognitive process”. Claiming that a given task captures a given elementary process, researchers incorrectly transfer the task intended requirements, as typically defined by the task instruction, onto their interpretation (naive model) of the information flow during solving the task, based on coarse-grained psychological concepts. At the same time, theoretical results from computational modeling in psychology and neuroscience show that this information flow needs to be described in terms of much finer-grained entities and their transformations. The picture is also complicated by the fact that the models proposed so far largely differ in how they describe such flow on the fine-grained level. 

For example, the well-known Stroop task is typically defined in psychometrics as an attention-control task, or a response-inhibition task, which requires blocking word reading while focusing attention on color naming (as asked by the task instructions). In computational cognitive neuroscience, a number of Stroop-like task models have been developed, which assume entities that can hardly match the above high-level description, including such terms as “energy”, “utility”, “activation”, “dimensional uncertainty”, and “inhibitory conductance”, to name only a few. Moreover, these models assume differing mechanisms responsible for effective color naming, including activation spread among concepts, lemmas, and word forms (Roelofs 2003), action utility learning (Lovett 2005), reinforcement learning (Holroyd et al. 2005), conflict adaptation (Botvinick et al. 2001), conflict-based Hebbian learning (Verguts and Notebaert 2008), contingency learning (Schmidt 2013), and outcome-action prediction (Alexander and Brown 2011) as well as some combination of these (Chuderski and Smoleń 2016). No model assumes explicitly the processes of word-reading inhibition and attention control over color naming. Moreover, no model assumes one central controlling process of attention control, but rather a complex interaction of diverse information flows (with adaptation and learning being important components). Finally, typical models include entities occupying various levels of abstraction, such as low-level associations (e.g., connection weights) linking higher-level objects (e.g., lemmas, production rules). As most of these models quite validly predict data from Stroop-like task experiments, we should accept that at least to some extent they capture the neurocognitive machinery generating the resulting behavior. Yet, they definitely do not match the naive models developed in psychometrics. So, it is fair to say that we do not know yet what exactly our minds do when they perform the Stroop task and multiple other ECP (e.g., the anti-saccade and vigilance) tasks; therefore, any unitary psychometric interpretation of them would be dubious.

An analogous situation pertains to working-memory tasks, with the one exception that they are even more complex than attention control tasks. Actually, from a perspective of cognitive modeling, a, let’s say, complex-span task (e.g., the operation span, which requires verification of a series of arithmetic equations and later recalling their solutions in a serial order) is comparably complex, as are some reasoning tasks presumably tapping fluid intelligence (e.g., number series, Latin square task, etc.). Moreover, there is an ongoing debate about what limits WMC: is it the number of available slots to maintain separate memory objects (Vogel et al. 2001); the number of the objects’ features that can be concurrently maintained (Fougnie and Alvarez 2011); the number of relations among objects and features (Clevenger and Hummel 2014); the size of the entire structure of such relations (Brady et al. 2011); some continuous resource that can feed memory representations (Bays 2015); interference among overlapping memory representations (Oberauer and Lin 2017); or an inability to desynchronize too many dynamic oscillatory patterns (Horn and Usher 1991; Raffone and Wolters 2001), to name only a few accounts. One consequence of the low consensus on the actual mechanisms underlying WMC (see Cowan 2022; Oberauer et al. 2018) is the fact that quite-distinct tasks have been applied to measure one and the same WMC construct (see Wilhelm et al. 2013), as well as one and the same working-memory task (e.g., the change-detection task; Vogel et al. 2001) being used to reflect many constructs beyond WMC, such as iconic memory (with rapid stimuli presentation; Sligte et al. 2010) and attention control (with a need to ignore some stimuli; Draheim et al. 2021). Due to our poor understanding of WMC, even the strong WMC–intelligence correlations help us little in advancing a theory of fluid intelligence. 

Third, it seems that the fact that correlational studies are correlational has been neglected when researchers draw conclusions from the correlations they observe. Even though each statistical handbook notes that correlation between X and Y can, in principle, be caused either by X or Y, or by their reciprocal interaction, as well as by another (typically unknown) variable Z (or more such variables), a superficial simplicity of ECPs relative to intelligence tests (shorter trials and simpler stimuli) as well as an implicit but ill-conceived reductionist stance has led many researchers to draw causal conclusions that intelligence varies because of ECPs. However, given the findings in cognitive modeling and cognitive neuroscience, it is likely that both X and Y are caused (or, better to say, modulated; Buzsaki 2006) by a large number of Zs—which can be understood as parameters of the cognitive system (Oberauer 2016), in part emerging from the underlying structural and functional brain architecture (Barbey 2018; Haier 2016). It is likely that intelligence tests can capture a larger number of such parameters, or capture them more reliably, than can tasks intended to capture ECPs. So, even though for most researchers it would sound heretical, instead of ECPs underpinning intelligence, it is equally plausible that it is intelligence, understood as a set of neurocognitive parameters validly captured by fluid intelligence tests, which translates onto the scores on ECPs (as suggested by Spearman 1927). At least, current psychometric studies can say little on the causality underpinning ECPs and intelligence.

To summarize this section, since neither experimental manipulation of individual intelligence levels nor fine-grained measurement and interpretation of the neurophysiology underlying these levels are possible at the current stage of scientific development, most research on fluid intelligence has had to resort to psychometric analyses of the correlational patterns between intelligence tests and various cognitive tasks. This is an inevitable research tool that has provided a highly informative (even though far from conclusive) “map” of relationships between cognitive constructs. However, this very tool seems strongly limited in the depth of fluid intelligence explanation it can yield. 

In the next sections, I shortly review two alternatives, more process-oriented approaches to developing a theory of fluid intelligence, which may offer insights beyond those offered by the psychometric approach. The first approach examines experimentally the properties of established fluid intelligence tests, trying to discover the crucial requirements of these tests—what minimal cognitive task is sufficient to capture the same variance in fluid intelligence that is captured by the tests used to date. It seems that little is required—just to validly represent a relatively simple relation. The second approach analyzes neurocomputational models of deductive and inductive reasoning in order to identify the sources of intrinsic limitation in representing relations. 

## 4. What Is Needed for a Task to Become a Fluid Intelligence Test?

Typical fluid intelligence tests involve identifying abstract rules governing relatively complex figural stimuli patterns and selecting the response option that best matches these rules. Probably the most widely applied test is Raven’s Advanced Progressive Matrices (RAPM; Raven et al. 1983), which presents the 3 × 3 matrices of geometric patterns, with a bottom-right pattern missing. RAPM requires inducing this missing pattern from the structure of the row- and column-wise variation among the remaining patterns, including permutation, increase in number or value, and logical relations such as AND, OR, and XOR. Depending on the type and number of rules (Carpenter et al. 1990) and the number of figural elements (perceptual complexity; Primi 2002), accuracy on consecutive RAPM items decreases from 90% to 10%. However, which RAPM features make it such a good fluid intelligence test (i.e., correlating so strongly with other such tests)? When analyzed in more detail, RAPM involves at least: abstracting the key geometric transformations from the perceptual input, which first must be identified (not easy in the case of overlaid complex figures); discovering the rules governing these transformations; constructing the missing element and/or inspecting the response options in search of a cue; actively maintaining the rules; and, finally, comparing the most plausible options in order to select the correct one. Are all of these processes (and perhaps others) necessary? Although there are only several reliable studies addressing this question, the answer is no: most of the above requirements are completely dispensable when measuring fluid intelligence.

Crucially, discovery of rules seems irrelevant for capturing fluid intelligence. Notably, in many other established fluid intelligence tests, including geometric analogies, paper folding, and necessary arithmetic operations (all very close to RAPM in the multidimensional scaling model of Snow et al. (1984)), the rules are trivial or revealed to participants. In line with this, in a convincing study, Carlstedt et al. (2000) administered three fluid intelligence tests in either the blocked order (one test applied after another) or with all the test items mixed. They assumed that in the blocked order the participants quickly discovered and learned all the rules required by a given test due to the homogeneous sequence of items, whereas in the mixed order discovery of the test rules was much more demanding, as the rules varied enormously. However, the blocked order, in which the participants already knew the rules for the middle and late items of each test, yielded higher loadings on the general intelligence factor than did the mixed item order.

Also studies of RAPM did not find that rule discovery matters. Loesche et al. (2015) applied RAPM either in a typical administration requiring rule discovery or after intensive training on rules, finding that in two out of three experiments the correlation with WMC (a proxy for cognitive ability in that study) increased after rule training (in the third experiment, the correlation was comparable). Thus, the need to discover rules might distort fluid intelligence measurement instead of improving it (see also Levacher et al. 2022). Although an initial work (Wiley et al. 2011) reported stronger RAPM item-wise correlations with the complex-span task, when the specific combination of rules was used for the first time throughout the RAPM test, compared to when it was repeated, suggesting a role for rule discovery, these correlations were overall very weak. Two later studies that observed stronger correlations (due to using the WMC factor instead of a single task) reported comparable correlations for new vs. old rule combinations (Smoleń and Chuderski 2015; Little et al. 2014). Finally, using a design that prevented potential confounds, Harrison et al. (2015) found that the correlation with WMC is actually higher for the old-combination items than for the new ones. Therefore, rule discovery seems to contribute little to fluid intelligence measurement.

These findings were supported by the statistical models that separated the item-position effect, assumed to reflect the learning of test rules from item to item, from the “pure” reasoning ability (i.e., with the item-position effect eliminated). Both for RAPM (Lozano 2015) and another fluid intelligence test (Schweizer et al. 2018), the item-position effect was not related significantly to other markers of cognitive ability. Even more, some studies (Hayes et al. 2015; Lozano and Revuelta 2020) suggested that the item-position effect in RAPM is not related to rule learning at all but mainly reflects more basic practice effects of optimizing perceptual and spatial strategies in the test. 

Loesche et al. (2015) observed, additionally, that after rule training the participants more often used the constructive strategy during coping with the RAPM items, as opposed to the response-elimination strategy (see Bethell-Fox et al. 1984; Vigneau et al. 2006). In the former strategy, the participants analyze the matrix trying to fully reconstruct the missing pattern and then look for its potential match among the response options. In the latter strategy, the participants develop only a partial pattern using the most salient cues and then use it to eliminate the most obviously incorrect options (and select from the remaining ones), toggling back and forth between the matrix and the response options. Therefore, large perceptual complexity and substantial variation in response options (a case of RAPM) actually can help to bypass the presumed cognitive requirements of the test, by facilitating the use of perceptual cues and simpler heuristics that allow to choose correct solutions above chance. Consequently, the test variants in which correct and incorrect response options were hardly distinguishable (Arendasy and Sommer 2013; Chuderski 2015b; Jarosz and Wiley 2012), or the responses had to be constructed from scratch (Becker et al. 2016), showed increased validity as fluid intelligence measures. In consequence, perceptual complexity and the number of response options contributes little—tests lean on perceptual content and excluding response options (so responses have to be construed; e.g., Jastrzębski et al. 2022; Thissen et al. 2018) seem to promote the uniform solution strategy and capture fluid intelligence more precisely (Levacher et al. 2022). 

Besides the perceptual complexity and response-option diversity of a fluid reasoning test, it is interesting what level of the problem’s scope and abstraction is actually required to tap fluid intelligence. Chuderski (2019) examined these questions in a series of six experiments using the transitive-reasoning task (Goodwin and Johnson-Laird 2008). By systematically simplifying the task, it was tested how trivial and concrete variants of it can still be apt measures of fluid intelligence (see Shokri-Kojori and Krawczyk 2018). The original task variant presented an abstract problem: “Three pairs of objects bound by relations ‘<’ or ‘>’ unequivocally define the monotonic linear order of four symbols. Organize the symbols in your mind into the valid order”. For example, the pairs could be “A > B”, “C < B”, or “C > D”, and the response to be selected could be either “D < A” or “D > A”. To respond correctly (“D < A”), all the pairs had to be integrated. This variant yielded low accuracy and strongly correlated with the fluid intelligence factor (*r* = .55). However, this correlation remained strong (*r* = .67) in a variant in which the participants were not given an abstract instruction to organize the symbols into the linear order, but only to decide which relation with the reversed symbols was the same as the relation in one of presented pairs (e.g., “B > A” or “B < A”?). The correlation held strong (*r* = .58) when no concept of relation was mentioned, but the participants were just asked to identify exactly the same pair as one presented (e.g., “A > B” or “A < B”?), and dropped only a little (to *r* = .46) when the task was to simply match the three-symbol string including a middle slash (e.g., does “A/B” match “A/B” or “A\B”?). The correlation only disappeared when the task was to match unbound pairs of symbols (is “AB” the same as “AB” or “BA”). It was concluded that full-blown abstract reasoning is not needed to capture fluid intelligence, and as little as a single trivial binding of simple information in the mind is critical.

These findings were compatible with the results on the so-called relation-monitoring task developed by Oberauer (1993), which required responding when the stimuli on the screen satisfied a simple predefined relation. In one task variant, people observed a constantly changing matrix of symbol strings and decided whether the three strings in one row, column, or diagonal line ended with the same symbol. The task, thus, required identifying relations among the symbols, while imposing relatively low storage requirements (all information was constantly available on-screen) and involving no form of reasoning (information did not need to be transformed in any way). Despite the task’s simplicity, several of its variants have been shown to capture fluid reasoning comparably to the hallmark reasoning and the working-memory tests (Bateman et al. 2019; Chuderski 2014; Jastrzębski et al. 2020; Oberauer et al. 2008). 

Finally, Jastrzębski et al. (2020) found that the factor that loaded the above mentioned task of comparing the “>” and “<” relations, the relation-monitoring task in which three symbols in a row or column had to be mutually different, as well as a novel simple task that required mapping two nodes between two structurally isomorphic but perceptually different graphs (Jastrzębski et al. 2022), was statistically indistinguishable from the factor that loaded typical fluid intelligence tests such as RAPM, Cattell’s CFT-3, and figural analogies. The two factors shared over 90% of variance, suggesting that the rank order of fluid intelligence can be reproduced with tasks devoid of perceptual complexity, rule discovery, abstraction, and multiple-response alternatives. Actually, these tasks involved neither complex rules nor multiple-rule integration, required no inference steps, and captured faster cognitive processes (response delivered in several seconds), as compared to typical matrix and analogical-reasoning tests (up to a minute required for a response). In their essence, each of these tasks required the processing of a single predefined relation that bound simple elements, such as symbols with their positions and letters with the “<” or “>” relation sign, as well as nodes and arrows in a graph. This is an important clue regarding what fluid reasoning can be about.

## 5. Computational Models Which Process Structures and Relations

Another source of insight on what is crucial for fluid intelligence comes from the computational models of structured information processing developed in cognitive neuroscience and cognitive psychology. In cognitive neuroscience, the rhythmic activation of neuronal groups encoding task elements was proposed as a mechanism underlying the brain processing of ordered and structured information. Probably the most widely cited iEEG study of the rat brain (O’Keefe and Recce 1993) showed that as a rat approached food, it used the subsequent firing of distinct hippocampal neurons in the gamma band, synchronized with the rat’s theta rhythm, to encode the consecutive steps on its route. Such a phase precession has recently also been reported for people (Qasim et al. 2021). Siegel et al. (2009) showed that a monkey was able to remember two pictures in the correct order, only if the sequential spiking pattern was present in its frontal cortex and the order of the spikes matched the order of presentation of the corresponding pictures (for recent analogous evidence regarding humans, see Bahramisharif et al. 2018).

Inspired by O’Keefe and Recce’s (1993) findings, Lisman and Idiart (1995) developed a computational model in which the lists of items in human short-term memory were encoded analogously, as rats encode consecutive locations—by a sequence of gamma cycles desynchronized by a global inhibitory signal, which forced each item representation to peak in a distinct phase of the theta cycle (see also models by Chuderski et al. 2013; Horn and Usher 1991; Koene and Hasselmo 2007; Raffone and Wolters 2001). The model showed that only a few elements can be held in order at one time, due to intrinsic limitations of neural oscillatory synchronization and desynchronization. 

Recent EEG (see Sauseng et al. 2019) and transcranial stimulation research (see Hanslmayr et al. 2019) supported this category of short-term-memory models. Analogous evidence for phase synchronization was reported for directing visual attention to consecutive objects (see Jensen et al. 2021). Even for response latency and hit rate, research showed that rhythmic asynchronous variation (so-called behavioral oscillations) can be aligned to items (Pomper and Ansorge 2021). Overall, the studies suggest that the brain encodes structures by dynamically organizing activation patterns in time, including the coupling of subsequent elements to phase (Cohen 2011).

In cognitive modeling, multiple models of problem-solving and reasoning assumed that representing and transforming relations, as well as mapping their structures across situations, is the core process (called *structure mapping*; Gentner 1983) leading to valid solutions and conclusions. These models described two tasks most strongly involving fluid reasoning (explicitly called *relational reasoning*; Holyoak 2012): analogical reasoning (Forbus et al. 1994; Halford et al. 2010; Keane et al. 1994) and inductive reasoning in matrix problems (e.g., the RAPM test; Carpenter et al. 1990; Lovett and Forbus 2017). Crucially, a group of reasoning models represented the key relational structures using rhythmic patterns of activations (see Shastri and Ajjanagadde 1993), providing convergence with the above-mentioned cognitive neuroscience studies on short-term memory and attention. 

**Figure 1:**
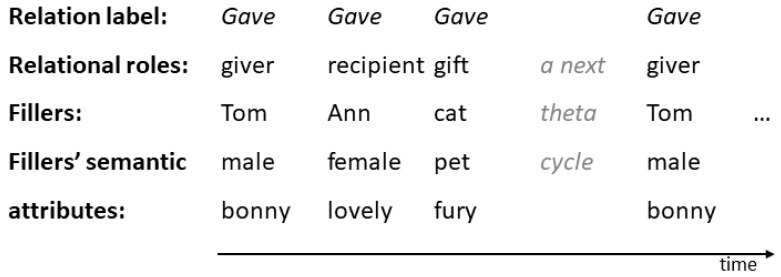
An example of temporal pattern of bindings in LISA’s representation of the relation instance *Gave* (Tom, Ann, cat).

For example, the LISA model of analogy-making (Hummel and Holyoak 1997, 2003) mapped and transferred the relations and their arguments between a familiar and a novel situation by means of discovering the structural and semantic correspondences between the two situations. LISA’s most important feature was that relations and their arguments had to be represented in the model’s working memory, which was limited to several role–object pairs. More complex relations had to be divided into smaller fragments (mapping was incremental). Each role of a relation was a distinct oscillation, and an object was bound in phase to the role it played. In contrast, the relation’s predicate oscillated for the total time of oscillation of all its pairs, binding them into the complete relation (see Figure 1). The relation cycle was associated with the theta rhythm, while the cycles for particular pairs reflected gamma oscillations (Knowlton et al. 2012). With more capacious working memory, LISA was able to process more complex analogies. The model was also adapted to explain the development of relational reasoning in children (Doumas et al. 2008).

As LISA has never been used for explicit simulations of the individual differences in reasoning performance, Chuderski and Andrelczyk (2015) developed an oscillatory model of a figural analogies test in which variation in the parameters governing the oscillations of bindings between consecutive figures and respective geometric transformations allowed for reconstruction of the distribution of analogical reasoning scores in the human sample, including the types of errors made by high- vs. low-performing participants. Rasmussen and Eliasmith (2014) developed a model, based on a spiking-neurons architecture, which used the dynamic patterns of activity to model reasoning on RAPM. This model simulated performance differences between younger and older participants. All these models constitute the proof of concept that representing relations with flexible patterns of bindings can both explain the processing in the fluid (i.e., relational) reasoning tasks and the individual differences therein.

## 6. Fluid Intelligence and Relational Representations 

On the basis of the findings described above, I propose that *fluid reasoning can amount to representing in the mind the key relation(s) for the task at hand in a valid and robust way*. Such relations could be encoded in the brain by an asynchronous pattern of a required number of correct role–filler bindings. I start to elaborate the above proposal by qualifying more precisely its main three elements: relations, valid, and robust.

*Relation* is defined as a labeled, ordered list (tuple) of arguments. A label identifies a relation and allows other entities to refer to it (e.g., a relation can be an argument for some other relation). In cognitive science and psychology specifically, arguments are more commonly called relational roles, and their values are called fillers (Doumas and Hummel 2005; Halford et al. 2010; Holyoak 2012). Each relational role is grounded in the human conceptual system, which provides knowledge on what fillers can play that role and what they can “do”. A label is associated with a relation’s intension. A relation divides the Cartesian product of all possible fillers that can be assigned to the roles in the relation into the subset, for which the relation is satisfied (“true”), and its complement, for which the relation is not satisfied (“false”). An ability to represent relations in the mind allows humans to abstract from perceptual and semantic properties of fillers (such properties can be misleading in abstract, formal reasoning; see Markman and Gentner 1993), supporting the compositionality and productivity of human thinking (Hofstadter 2001). 

However, in order to do so, mental representations of relations need to be *valid*, that is, they must include the correct bindings between respective fillers and their roles as well as preserve these bindings during consecutive mental operations. First, fillers need to satisfy the categorial constraints for particular roles (Keane et al. 1994)—for instance in the relation *Gave* (giver, recipient, gift), the role of giver assumes a person or a group. The relation’s instance *Gave* (Tom, Ann, cat) clearly satisfies this constraint; but, in a creative story on a magic cat in which the constraints can be violated, it would also be acceptable that *Gave* (cat, Tom, Ann). However, as many fluid intelligence tests are semantically lean (include meaningless symbols or shapes), the above categorial constraints help little in processing relations. Second, and more important, the relation representation must be structurally consistent within the entire system of relations a person holds (Halford et al. 2010), so when considering the relation *Received* (recipient, giver, gift), the actors and objects must play the corresponding roles as in *Gave*, that is, *Received* (Ann, Tom, cat). Third, the categorial and structural properties of relations together allow for making multiple inferences. For instance, knowing that “A is above B” and “C is below B”, one can infer that “A is on top”, “C is at bottom”, “B is between them”, etc. 

Simple fluid intelligence tests may capture fluid reasoning so aptly because they require a valid relation representation. For instance, in the relation-monitoring task, such a representation must include only the last symbols bound correctly to the consecutive matrix positions (e.g., first, second, third column), such as *Row1* (Ψ, Φ, Ω). Following the structural consistency principle, it needed to be transformed (in a process sometimes called relational integration; Oberauer et al. 2008) into some form of representation that explicitly reflects three symbol differences, for instance *Diff* (Ψ ≠ Φ, Φ ≠ Ω, Ψ ≠ Ω).

The condition that relation representation has to be *robust* means simply that this representation has to be accessible, as long as it is required during coping with a task. For instance, the relation *Row1* cannot break down before the relation *Diff* is construed (but can be dispensed afterwards). Failure to access the key relation, either because of failure to construe it or to maintain it after construal, likely leads to a failure on the task.

The power to represent relations in a valid and robust way potentially provides an individual with crucial flexibility in dealing with cognitive tasks, because relational representations can encode arbitrary knowledge structures of any form. Especially, they enable novel structures, including ones which are not possible in the real word (“a mouse bigger than an elephant”), underlying innovative and creative thinking. This very flexibility stems from the fact that relational roles can, in principle, be paired with any fillers (see Doumas and Hummel 2005). Moreover, dynamic bindings can potentially encode diverse types of structures (e.g., lists, stacks, trees, networks), because relational roles can easily define particular placeholders in a specific structure (“a node”, “a root”, etc.). Finally, the intrinsic trade-offs between the size (many asynchronous bindings) vs. stability/precision (only one or two bindings, but each of them binding together rich information) can be potentially resolved by adapting the internal organization of bindings to the requirements of a task. For instance, in some situations (e.g., a change-detection task) it is more important to encode a large number of objects, even at the risk of losing some of them, while in some other situations (e.g., attention-control tasks), it is crucial to focus on a single maximally undistorted representation for as long as possible (see the adjustable-attention hypothesis by Cowan). Dynamic bindings seem to allow all that flexibility.

The effective maintenance of relation representations may drive individual differences in fluid reasoning, because evidence from cognitive neuroscience and cognitive modeling suggests that processing relations in a valid and robust way is very difficult for such a biological system as the human brain. Specifically, if the oscillatory models of working memory and reasoning are right, the relation representation requires the maintaining of an arbitrary and precise pattern of dynamics: representation of a given relational role and corresponding filler must be maintained in synchrony, while consecutive role–filler bindings must be active asynchronously. Both data from brain recordings (Bahramisharif et al. 2018; Qasim et al. 2021) and computational models (Chuderski and Andrelczyk 2015; Usher et al. 2001) suggest that maintaining such patterns is a demanding process that may become unstable with three or more bindings. The individual differences in fluid intelligence may, thus, stem from whether one succeeds or fails to process simple relations in a valid form (e.g., without missing or mixing roles and fillers) for the entire interval during which this relation is needed.

The above proposal should not be treated as a novel theory of fluid intelligence, just as a working hypothesis. In the present state of cognitive (neuro)science, we still await more precise data and models on how humans process relations, which are not easily extractable from the black box of the human mind. Even though psychometrics (new simple fluid intelligence tests), brain imaging (a growing evidence for the role of rhythmic neural patterns in cognition), and cognitive modeling (development of the models of relational reasoning yielding insight into the underlying mechanisms) provided initial cues for the conceptualization of fluid intelligence in terms of flexible relational representations, future research is needed to develop a causal theoretical model explaining how dynamic patterns in the brain could translate into relational representations, which themselves would translate into the processing of cognitive tasks (including fluid intelligence tests) that, in turn, translates into academic, professional, socioeconomic, and life status. However, even not being such a theory, the current proposal helps to purify the fluid intelligence construct and develop new ways of its scientific pursuit.

Even though a direct method to observe how relations are represented in the brain is still lacking (but we can already identify simple list structures encoded by phase precession (Qasim et al. 2021)), several kinds of less direct evidence could either support or undermine the role of relation representation for fluid intelligence. On the psychometric level, as the work by Jastrzębski et al. (2020, 2022) can count only as initial evidence, it should be systematically examined whether novel relation-processing tests can substitute for the traditional tests in the fluid intelligence measurement (e.g., explaining the same variance, surpassing competitor predictors such as working-memory tasks) and can equally strongly predict phenomena known to relate substantially to fluid intelligence (e.g., academic achievement, learning). On the cognitive level, computational models that allow variation in relation representation effectiveness should be able to replicate main findings from the reasoning ability literature (distributions of scores, pattern of errors, interrelations between various tasks, developmental patterns, etc.), even though successful simulations can never act as a decisive proof. On the neural level, assuming that temporal patterns of bindings are encoded via their coupling to the phase of some brain rhythm, certain parameters of coupling (yet to be identified) are expected to more strongly predict scores on both simple relation-processing and typical fluid intelligence tests, as compared to alternative brain markers (for initial evidence pertaining to the delta-gamma coupling see Gągol et al. 2018). Finally, the holy grail of intelligence research is cognitive training capable of increasing individual intelligence levels; however, a recent second-order meta-analysis refuted that visible far-transfer effects can be achieved by working-memory training (Sala et al. 2019). The plausibility of the relation-representation hypothesis would be strongly supported if, instead, the training programs based on trivial relation-processing tasks resulted in persistent increases in fluid intelligence, operationalized at the latent level (for initial evidence of a successful relation processing training in adolescents, see McLoughlin et al. 2020).

Similar relation-based proposals present in the literature as well as their differences with the current proposal need to be highlighted. A concept of *relation* appeared a century ago in Spearman’s (1927) idea of *eduction of relations and correlates* (analogous *discriminating and perceiving relations* was also considered by Cattell (1943)). However, the psychometric evidence cited above suggests that neither identification of the valid relation when the values of its arguments are known (rule discovery) nor assigning the valid values to the arguments for the known relation (relation instantiation) is crucial for capturing fluid intelligence. Instead, a more basic process of relation representation is considered here: even when both the relation and its arguments are provided by the task, it is still demanding for the brain to maintain the resulting pattern of bindings for a required duration. Moreover, this representation is necessary for processes that are conceptually simpler than eduction of relations, such as bare validation of a relation—checking whether the current assignment of fillers to roles satisfies that relation, or even just reporting a relation—recalling an ordered list of elements in the complex-span task. 

As compared to Oberauer’s (1993; Oberauer et al. 2008) idea of *relational integration*, and an analogous conception of *construction of relational representations* by Halford et al. (2015), which saw the crucial source of fluid intelligence differences in the process of integrating basic elements of relations into more complex relational structures, which may succeed or fail, the current proposal focuses primarily on the very maintenance of those basic elements, as it can be demanding even in the absence of the need for relational integration. However, Duncan et al. (2017) showed that a fluid intelligence test with reduced rule integration (each rule could be processed separately) became feasible even for low IQ participants, implying the key role of relational integration (compositionality, in their terms). It is likely that both these sources of variance, mutually entangled to a large extent, do concurrently contribute to fluid intelligence, as suggested by initial research that contrasted these two sources (Shokri-Kojori and Krawczyk 2018). Definitely, more detailed research is needed here. 

As to Halford et al.’s (1998) idea of *relational complexity*—the maximum number of independent variables or relation arguments that one can grasp simultaneously without segmentation and chunking, which limits the reasoning process one can handle (Andrews and Halford 2002), the current proposal is also somewhat more basic: according to it, individual differences in fluid intelligence can also emerge from failures to robustly maintain relations lower in relational complexity than the maximum possible complexity of relation an individual can process. 

Finally, relational frame theory (Hayes et al. 2001), which describes how relational processing and behavior can emerge from operant conditioning (so-called *arbitrarily applicable relational responding*), seems compatible with the current proposal, yet it is formulated in a much more abstract way. Moreover, this theory primarily pertains to the development of relational processing, instead of the adult differences in it. 

In short, the present proposal claims that neither the process of integrating the bindings into the complete relational representation nor the available relational complexity of such a representation are crucial for individual differences in fluid reasoning, even though they may contribute to some of its variance. By contrast, such differences primarily reflect the validity and robustness of the more basic role–filler bindings representing relations. Inability to maintain, validly and robustly, such bindings in the brain/mind, as a result, limits the construal and processing of relation representations, even simple ones. 

## 7. Conclusions

In this work, I focused on the importance of relation representation and processing for the construct of fluid intelligence. I argued that when inessential features of fluid intelligence tests are abstracted away, the process captured by these tests amounts to representing the key relation(s) in the valid and robust way. Based on my review of neurocognitive data and models, this ability may itself be rooted in the parameters determining the maintenance of arbitrary patterns of dynamic bindings linking consecutive arguments of a relation with the roles played by them. These arbitrary, dynamic bindings potentially allow substantial flexibility of (relational) thinking. How valid and robust representations of relations one can maintain due to valid and robust bindings, may determine a fluid intelligence level one displays. Validation of this proposal requires novel precise data and models, with the future integration of findings from cognitive neuroscience and psychology with computational models of working memory and reasoning. The proposal can potentially help to reconceptualize fluid intelligence towards a more process-oriented construct (theory), as compared to the traditional correlational approaches considered to date. Although such a complex phenomenon as fluid intelligence definitely cannot be reduced to a single factor, the idea of relation representation may purify this very phenomenon and stipulate new lines of research.

## Data Availability

No data associated with this work.
